# Extracellular Matrix is an Important Component of Limbal Stem Cell Niche

**DOI:** 10.3390/jfb3040879

**Published:** 2012-12-10

**Authors:** Hua Mei, Sheyla Gonzalez, Sophie X. Deng

**Affiliations:** Jules Stein Eye Institute, 100 Stein Plaza, Los Angeles, CA 90095, USA; E-Mails: Mei@jsei.ucla.edu (H.M.); s.gonzalez@jsei.ucla.edu (S.G.)

**Keywords:** extracellular matrix, stem cell niche, limbal stem cell, limbus

## Abstract

Extracellular matrix plays an important role in stem cell niche which maintains the undifferentiated stem cell phenotype. Human corneal epithelial stem cells are presumed to reside mainly at the limbal basal epithelium. Efforts have been made to characterize different components of the extracellular matrix that are preferentially expressed at the limbus. Mounting evidence from experimental data suggest that these components are part of the stem cell niche and play a role in the homeostasis of limbal stem cells. The extracellular matrix provides a mechanical and structural support as well as regulates cellular functions such as adhesion, migration, proliferation, self-renewal and differentiation. Optimization of the extracellular matrix components might be able to recreate an ex vivo stem cell niche to expand limbal stem cells.

## 1. Introduction

Corneal epithelium is constantly renewed by corneal epithelial stem cells, which locate primarily at the basal layer of corneoscleral limbus although central corneal epithelium is also reported to retain limited self-renewal capacity in some animal models [1]. The stem cell niche where the corneal epithelial stem cells, limbal stem cells (LSCs) reside is intensively investigated. The niche has been proposed to maintain the stemness and proliferation of the stem cells. One critical component of the stem cell niche is the extracellular matrix (ECM), which supports cell adhesion, transduces signals to cells through binding to membrane receptors, provides the mechanical strength and buffering space for tissue, and serves as media for intercellular communication. Components of the ECM in the ocular surface are discussed in this article including their potential functions and applications with a focus on the LSC niche.

## 2. Discussion

### 2.1. Collagens

Collagens are the most abundant proteins in mammals accounting for 25–30% of the body protein mass and are an essential component of epithelial basement membrane zone (BMZ). Collagens comprise 28 members in vertebrates [2] and each member is named using Roman numerals. All collagens are composed of three polypeptide α chains, which can be the same α chain to form homotrimers (*i.e*., Collagen III) or different α chains to form heterotrimers. Each α chain is named with an Arabic number followed by a Roman numeral which is the type of collagen it comprises. For example, Collagen I is composed of 2 α1(I) and 1 α2(I) chains. Collagen IV is also composed of 3 α chains, but there are 6 different types of α chains found in Collagen IV [3,4]. Efforts have been made to compare the expression patterns of different collagens between the central and limbal corneal BMZ in adult human and between fetal/infant and adult human cornea. The collagen members whose expressions are predominant in adult limbus or predominant in developing cornea and the limbus are possible candidate components of limbal stem cell niche.

Among the 28 collagen members from I to XXVIII, eight of them are reported to be expressed in adult human cornea and limbus. These include collagen III, IV, V, VII, XV, XVI, XVII, XVIII (Table 1). As there are six genetically distinct α chain for collagen IV, designated α1-α6 [3,4], the expression of collagen IV was categorized with more details on its α subunits in this table. Among all the collagen members and subunits shown in Table 1, only α1, α2 chains of collagen IV and collagen XVI have preferential expressions in adult limbus, whereas total collagen IV is expressed at similar levels throughout the cornea, limbus and conjunctiva [5]. Collagen XVI is preferentially expressed in the region below the BMZ of peripheral cornea and anterior limbus [5,6,7]. 

Expression of several collagens has been studied in fetal, infant and adult cornea. These include collagen III, IV, VII, XII, XVI, XVII and XVIII. Collagen III was expressed in the fetal epithelial BMZ, stroma and Descemet’s membrane; however its expression disappeared in the central cornea later during gestation [8], but present subepithelially in adult limbus [9]. It is unclear whether it is expressed in the conjunctiva. Only the α1 and α2 chains of collagen IV and collagen XVI are found to be preferentially expressed in fetal and adult limbus, and in fetal cornea and limbus. 

**Table 1:** Collagens expression in the basement membrane of adult human cornea, limbus and conjunctiva by immunohistochemistry.

Collagen	α chain	Cornea	Limbus	Conjunctiva	Reference
III		-	+	?	[9]
IV	α1	-	+	+	[5,7]
	α2	-/w	+	+	[5,7]
	α3	+	-	-/w	[5,7]
	α4	?	-	-/w	[5,7]
	α5	+	+	+	[5,7]
	α6	+	+	-/w	[5]
V		+	-	-	[5]
VI		-	-	-	[5]
VII		+	+	+	[5,7]
XV		+	+	+	[5]
XVI		-/w	+	-	[5]
XVII		+	+	+	[5]
XVIII		+	+	+	[5]

**+**: expression present; -: expression absent; -/w: negative or weak expression; ?: conflict/unconfirmed expressions.

The function of collagen IV α1 and α2 chains in stem cells is largely unknown. It is recently reported that their expression is increased during the adipogenic differentiation of human mesenchymal stem cells [10]. In limbal stem cells, some *in vitro* indirect evidence indicates that corneal epithelial progenitor cells preferentially bind to collagen IV. Collagen IV has been used to enrich the limbal stem cell population [11]. The rapid adhesion cells showed a less differentiated phenotype. These cells had enriched BrdU-label retention, higher expression of putative stem cell makers, minimum expression of differentiation markers, higher colony-forming efficiency and higher growth rate [11]. 

Moreover, collagen IV has been shown to serve as the substrate to induce embryonic stem cells differentiating into epithelial lineage cells. Mouse embryonic stem cells cultured on plates coated with collagen IV showed monolayer epithelium-like morphology which expressed keratin (K) 12, a corneal epithelial maturation maker, but not K14 which is expressed in the less differentiated corneal epithelium [12]. Similar differentiation was observed in human ES cells cultured on collagen IV in the conditioned medium from limbal fibroblasts [13]. Besides embryonic stem cells, murine vibrissa hair follicle stem cells can be induced to transdifferentiate into corneal epithelial-like cells on collagen IV or laminin (LN)-5 in the presence of conditioned medium from limbal fibroblasts [14]. In contrast, the hair follicle stem cells did not attach or grow on LN-1 or fibronectin (FN) coated plates [14]. These corneal epithelial-like cells expressed corneal-specific K12 and Pax6, and reduced expression of K10, an epidermal epithelial cell marker. 

Collagens, which have excellent biocompatibility, proper bio-degradation rate, low immunogenicity and can be structured into certain mechanical strength, are a very good material as corneal scaffolds. Type I collagen fibrils are induced to align orthogonally in a high magnetic field, which mimic the fibril organization of corneal stroma and the transparency can be greatly improved by adding proteoglycans [15,16]. Such scaffolds allow the *in vitro* construction of human hemi-corneas comprises of stroma and epithelium by seeding human keratocytes and limbal stem cell derived epithelial cells [15,16]. Collagen vitrigel which is made of type I collagen is used to culture epithelial cells, keratocytes and endothelial cells. Vitrigel possesses proper tensile strengths, thus makes it a potential scaffold for corneal stroma reconstruction [17].

The mechanical properties of the collagen scaffolds affect the growth and differentiation of limbal stem cells. Collagen gel made from rat-tail type I collagen was compressed to increase its mechanical stiffness [18]. Bovine limbal epithelial cells grown on compressed collagen I scaffolds demonstrated a more differentiated phenotype with a higher expression of K3 and lower expression of K14 than those grown on uncompressed collagen I [18]. 

Based on the preferential components of the specific collagens of the limbus, the following components may play an important role in maintaining the stem cell properties and might be beneficial for the *in vitro* expansion of limbal stem cells: collagen III, the α1 and α2 chains of IV, and XVI. Potentially, the mechanical properties of the collagen I scaffold can be further optimized to expand limbal stem cells *in vitro*.

### 2.2. Glycoproteins

#### 2.2.1. Fibronectin

Fibronectin is a ubiquitous ECM glycoprotein. It is present in a soluble form in plasma and synthesized by hepatocytes. It also exists in an insoluble form in tissue that is synthesized by fibroblasts and epithelial cells [19]. The FN protein is produced from a single gene but alternative slicing leads to 20 isoforms in human [20]. 

FN is preferentially expressed in the epithelial BMZ of human adult limbus and fetal cornea. In adult, it has the highest expression at both anterior and posterior of limbus while its expression in the cornea and conjunctiva is lower [5]. During development, FN appears briefly in the corneal epithelial BMZ and corneal stroma between 8 to 37 weeks of gestation [8]. 

Although there is no report of FN in the function of limbal stem cells, its potential roles in other stem cells have been investigated. FN mediates cell adhesion and promotes cell migration of human bone marrow stem cells [21]. It serves as a chemotactic cytokine responding to factors such as platelet-derived growth factor-BB and induces significant mitogenic activity for both rabbit and human mesenchymal stem cells [22]. In zebrafish, the absence of FN leads to the formation of impaired adherent junctions between myocardial precursors, which in turns leads to defective epithelial maturation/organization of the myocardial precursor cells [23].

#### 2.2.2. Laminins

Laminins are also major components of BMZ in both vertebrates and invertebrates. It is a heterotrimer protein composed of an α, a β and a γ chain [24]. To date there are five α, three β and three γ chains identified in vertebrates [24,25]. LN is also named after their sub-unit types. For example, LN-21, also referred as LN-2 in old nomenclature, is composed of α2, β1 and γ1 subunits. 

The expression patterns of different LN subunits in the BMZ of adult human corneas are summarized in Table 2. α3, α4, β3 and γ2 chains are expressed at comparable levels in central cornea, limbus and conjunctiva [5,6,26,27]. α1 and γ3 are more specific in the limbus than in the cornea or conjunctiva [5,6,26] whereas α2, α5, β1, β2 and γ1 are expressed in the limbus and conjunctiva [5,6,26,27]. 

**Table 2:** Laminins expression pattern in the basement membrane of adult human cornea, limbus and conjunctiva through immunohistochemistry.

Laminin		Cornea	Limbus	Conjunctiva	Reference
α	α1	?	+	?	[5,6]
	α2	-	+	+	[5,6,26,27]
	α3	+	+	+	[5,6,26]
	α4	?	?	-	[5,26]
	α5	?	+	+	[5,26]
β	β1	?	+	+	[5,6,26,27]
	β2	-	+	+	[5,6,26,27]
	β3	+	+	+	[5,6,26]
γ	γ1	?	+	+	[5,6,26,27]
	γ2	+	+	+	[5,6,26,27]
	γ3	?	+	-/w	[5,26]

**+**: expression present; -: expression absent; -/w: negative or weak expression; ?: conflict/unconfirmed expression results.

Besides their expression patterns in adult ocular surface, their expression patterns were also examined and speculated during development. Different α, β and γ chains were screened in infant and adult cornea. Those α, β and γ chains detected in the tissue had different combinations and gave rise to a list of LNs which were speculated to be present in the tissue. Among those LNs speculated, LN-321, -323, -421, -423, -521, -522 and -523 were proposed to be present in both infant and adult limbus but absent in infant and adult cornea. LN-211, -213 and -221 are present in adult limbus but absent in adult cornea and in infant cornea and limbus [26].

Some LNs are reported to facilitate cell adhesion, survival and migration of different stem/progenitor cells. LNα5 serves as the adhesion substrate for cell adhesion of 35–40% human bone marrow cells. Moreover, it facilitates the migration of human bone marrow progenitor cells stimulated by stromal-derived factor-1α (SDF-1α) [21,28]. It is also reported as a potent *in vitro* adhesive substrate for neonatal and adult keratinocytes. It increases the proliferation of neonatal foreskin keratinocytes and adult breast skin keratinocytes and stimulates keratinocytes migration *in vitro* [29]. When peptides containing globular domains derived from LNα1 and LNγ1 chains were incorporated into collagen hydrogel, neural stem cell survival has improved compared to the collagen hydrogel alone [30]. A LNγ1 deficient embryonic stem cell line can differentiate into cardiomyocytes; however, the differentiated cardiomyocytes lacked the BMZ and had impaired matrix deposition thus defective electronic signal propagation [31], indicating that LN γ1 may not be essential for the cardiomyocyte differentiation but is essential for the proper function of the differentiated cardiomyocytes.

Although some of the proposed niche-related LN subunits are suggested to facilitate stem cell adhesion, survival, migration and adequate formation of ECM as stated above, none of them has been tested for its function in limbal stem cells. Some other LNs, which have no preferential expression at the limbus or in the fetal cornea, have been examined for their *in vitro* application in corneal epithelial cells. LN-5 (LN-332) can serve as the substrate inducing the trans-differentiation of murine vibrissa hair follicle stem cells into corneal epithelial-like cells in the presence of conditioned medium from limbal fibroblasts [14]. It is also suggested that the proteolytic fragment of LN-5 γ2 chain may potentiate the outgrowth of limbal epithelial cells on intact amniotic membrane [32].

Eighteen LN deficient mice have been generated (summarized by [24]) and none of them shows obvious corneal defects although some of the knock-out mice, such as α3^-/-^, α5^-/-^ , β3^-/-^ and γ2^-/-^ show defects in the epithelium including skin blistering, defective hair follicle development and dental epithelium [33,34,35,36,37]. All these four knockout mouse strains are lethal either at embryonic stage or within 5 days after birth when the eyes are still closed. Whether there is any defect in the corneal epithelium is unknown.

#### 2.2.3. Vitronectin

Vitronectin (VN) is a glycoprotein circulating in the blood stream mostly as monomers or dimmers. When it enters the ECM, it forms multimers which bind to and incorporate into the ECM [38]. VN is highly expressed at the limbus and has minimum or no expression at the central cornea or the conjunctiva [5,39]. Human limbal epithelial cells grown on VN-coated plates gave higher colony forming efficiency and higher number of holoclones than the cells grown on uncoated plates [39], suggesting that it might help propagate the stem/progenitor cell population *in vitro*.

VN, however, is reported to induce differentiation of mesenchymal stem cells and cancer stem cells. Human mesenchymal stem cells undergo osteogenic differentiation when cultured on VN [40,41]. Cancer stem cells cultured on VN showed decrease expression of stem cell genes and modulated differentiation [42]. VN also serves as cytokines and induces significant mitogenic activity for both rabbit and human mesenchymal stem cells [22]. Whether VN functions through different mechanisms in different stem/progenitor cells and whether VN can serve as a niche factor for limbal stem cells needs to be examined carefully.

#### 2.2.4. Tenascin C

Tenascin C (TN-C) is the founding member of a family of vertebrate ECM glycoproteins which also includes other four isoforms, TN-R, TN-W, TN-X and TN-Y. All of these isoforms share a common primary structure composed of different motifs: the amino-terminal extreme with heptad repeats, the epidermal growth factor (EGF)-like repeats, the FN type III domain repeats and the carboxyl-terminal fibrinogen-like globular domain. Each of these motifs interacts with different group of ligands, such as cell surface receptors and other ECM components. Among the different TN isoforms, only TN-C has been shown to be alternative spliced [43]. 

Their functions are directly related to two opposite activities which reside in the native molecule, adhesion and de-adhesion, which are indirectly modulated by their interactions with other ECM components such as LNs and cell surface receptors. Among the cell surface receptors for TN-C are members of the integrin family, cell adhesion molecules of the immunoglobulin family and annexin II. TN-C can also bind to FN and chondroitin sulfate proteoglycans (CSPG) including perlecan (PC) and versican [43]. TN-C rich matrix has an impact on tissue resilience, enhancing tissue tension and stiffness, and modulating cell adhesion by promoting cell de-adhesion [44]. Accumulating evidence indicate a pivotal role of TN-C in cardiac injury, atherosclerosis, tissue remodeling, tumurigenesis and metastasis (reviewed in [45,46,47]. TN-C has been also associated as an extrinsic regulator for stem cell regeneration in various stem cell microenvironments, particularly in neural and hematopoietic stem cells [48].

In the developing embryo, TN-C displays a tissue and time specific expression. Particularly in the cornea, TN-C is highly expressed in human preterm corneas but its expression decreases in neonatal corneas [49]. Its expression in adult is restricted to the limbus [50]. Some studies have revealed the presence of mRNA variants in epithelium, stroma and endothelium of normal adult corneas but the proteins were not detected in either of them [49,51].

Like FN, the expression of TN-C is also upregulated during wound healing and then downregulated after tissue repair is completed. TN-C is localized around active ulcers in inflamed tissue and vascularized regions. It might modulate wound healing and cornea repair. Moreover, it is also present in areas in which the epithelium is detached from the underneath stroma, very close to the epithelium in the BMZ, indicating that TN-C may be synthesized by the epithelium [52]. The presence of TN-C has also been reported as late as three months after the primary wounding, indicating that TN-C, other than supporting migration and adhesion, may contribute in the subsequent remodeling of the cornea stroma. However, TN-C is not expressed in mature avascular scar tissue [53].

The search for function of TN-C has also been assessed through the generation of TN-C knockout mice. In the cornea, significant differences have not been observed in wound healing between wild type and knockout mice in linear incision injuries. In suture wounds induced by mechanical stress, TN-C upregulates the expression of FN and the lack of TN-C may impair stromal cell migration [54,55]. 

The alternatively spliced forms of TN-C may be important in the cornea development and remodeling mediated through functions such as proliferation, migration and differentiation. Moreover, the restriction of its expression to the adult limbus and developing corneas suggests its function in the self-renewal and differentiation of limbal stem cells by possibly providing a suitable matrix environment for the maintenance of the stem cell properties and guiding the differentiation towards corneal epithelium. The functions of TN-C are a consequence of its interaction with an important ECM component, the laminins, and with other cell surface receptors depending on the specific tissue.

#### 2.2.5. SPARC

SPARC (Secreted protein acidic and rich in cysteine) also known as BM40, or osteonectin is a highly conserved ECM glycoprotein consisting of three domains: an extracellular carboxyl-terminal extreme with two high affinity calcium-binding sites, a follistatin-like domain and an acidic amino-terminal extreme [56]. Although this glycoprotein was originally found to be a bone component [57], it has a widespread distribution in tissues and organs.

SPARC is predominantly expressed during embryonic development and adult tissues undergoing tissue remodeling or repair. SPARC is thought not to play a structural role but has been shown to regulate morphogenesis, disrupt cell adhesion and hence changes in cell shape, cell proliferation, migration and differentiation, and modulate cell-to-cell and cell-matrix interactions, wound healing and angiogenesis [56]. 

SPARC has also been proposed to regulate stem cell phenotype. For example, it has been shown that the absence of SPARC led to a decrease in the bone mass maybe due to an affected assembly or localization of growth factors which influence differentiation of mesenchymal stem cells to osteoblasts [56]. All of these functions are mediated through the interaction of SPARC with several ECM molecules including thrombospondin 1 (TSP 1), VN, nidogen, collagens (types I, II, III, IV and V) and with growth factors, such as PDGF and vascular endothelial growth factor (VEGF). 

In non-pathological or normal human corneas, SPARC is weakly expressed in the epithelial BMZ in both central cornea and limbus of infant and adult [26]. Their expression in quiescent corneal stroma has not been reported. The authors suggest that the horizontal heterogeneity along the epithelial BMZ both during the embryonic development and postnatal life could reflect the need of the limbal stem cells and transient amplifying cells to maintain a special structural organization of SPARC to preserve their undifferentiated state. Furthermore, they propose that this glycoprotein could be a marker of limbal stem cells as it has been shown to co-localize with some of the limbal stem cell markers, such as ATP-binding cassette sub-family G member 2 (ABCG2) and p63.

The role of SPARC in cornea wound healing has been studied using an *in vitro* stromal repair and wound model in bovine [58]. These authors observed that in this system the secretion of SPARC was higher when transforming growth factor-β (TGF-β) and serum is added to keratinocytes in culture made myofibroblastic. Conversely, SPARC secreted by epithelial cells induces contraction in stromal fibroblasts thus mediating epithelial-stromal interactions [59]. The transiently high expression of SPARC during cornea repair as well as in other pathological conditions suggest that SPARC might be regulated by different growth factors and cytokines, such as EGF, TGF-β, interleukin 1β (IL-1β) and PDGF-BB [60,61]. However, Shimmura and co-workers observed that limbal fibroblasts *in vitro* and *in vivo* secrete higher levels of SPARC compared to corneal fibroblasts suggesting a potential functional role of SPARC in homeostasis of the limbal stem cell niche [62]. 

#### 2.2.6. Nidogen

Nidogen also called entactin is a ubiquitous BMZ glycoprotein composed of three globular domains connected by a thin segment. It forms a light complex with LN, interacts with itself as well as type IV collagen and FN [63]. It is thought to play a role in cell adhesion and influences cell polarity and migration. It has also been proposed to play a role in cornea homeostasis and wound healing [63].

Nidogen has two isoforms, nidogen 1 and 2. Isoform 2 is highly expressed in the limbal and conjuntival BMZ but not in the cornea in both infant and adult [5]. This regional difference is thought due to the degree of differentiation of the epithelial cells and the higher expression of nidogen may be an indicative of its role in the stabilization of the BMZ.

To further investigate the role of nidogen in cell adhesion, different *in vitro* models have been used. Mishima and co-workers [64] showed that cell adhesion is enhanced in rabbit corneal epithelial cells in proportion to both the incubation period and the nidogen concentration when it is added to LN-coated plates. However, when collagen type IV and FN-coated plates are used, nidogen does not affect cell attachment. Thus, nidogen 2 may be an important component of the BMZ together with collagen IV and laminin to further optimize the expansion of limbal stem cells *in vitro*.

#### 2.2.7. Thrombospondins

Thrombospondins (TSP) belong to a family of multifunctional glycoproteins of the ECM that includes at least five members. Every protein in this family displays a distinct tissue distribution [65]. In the normal human cornea, the expression of two of these glycoproteins has been observed. TSP1 expression is restricted to the corneal BMZ and it is thought to play a role as a potent inhibitor of angiogenesis. TSP4 is focally present in the BMZ of peripheral cornea, limbus and anterior conjunctiva [5]. The role of TSP4 in the cornea is still poorly understood but the distribution of this glycoprotein may indicate a function in the maintenance of the stem cell microenvironment. 

### 2.3. Proteoglycans

#### 2.3.1. Agrin

Agrin is an ECM component that belongs to a diverse family of heparan sulfate proteoglycans (HSPG), also including syndecans, glypicans, perlecan (PC) and collagen XVIII. HSPG regulate many signal transduction pathways in development, including Wingless, Hedgehog, fibroblast growth factor (FGF), and TGF-β. Agrin plays a role in normal homeostasis during development, tissue morphogenesis and wound healing. Agrin null mice are embryonic lethal [66]. Moreover, it is a key component of BMZ in many tissues [67] and it is involved in establishing tissue barriers and regulating muscular synapsis [68]. Agrin plays a crucial role in the short-term hematopoietic stem cells development by mediating the crosstalk between the hematopoietic stem cells and their stromal cells [69]. 

Agrin has abundant expression in the eye. Overexpression of agrin in mice leads to ocular dysgenesis including anophthalmos, microphthalmos, adhesion of iris and lens to the cornea, and coloboma of the optic stalk by repressing Sonic Hedgehog signaling [70]. Agrin is absent or weakly expressed in central cornea but its expression slightly increases in peripheral cornea and conjunctiva. The highest expression is found in the limbus in a continuous or patchy pattern which coincides with regions where limbal stem cells reside [5]. 

To study agrin’s function in the eye, different mice models have been developed. It has been shown that the deletion of agrin causes neonatal mortality due to the failure of the neuromuscular junction formation. However, the overexpression of agrin promotes ocular dysgenesis. In the cornea, agrin has been proposed to participate in cell proliferation, migration and differentiation due to its co-localization with limbal stem cells and stem cell markers including ABCG2 and p63 [5]. Thus a proper cell adhesion is a crucial determinant in the homeostasis of the stem cell niche and guiding the differentiation of these stem cells during regeneration of the cornea epithelium. 

#### 2.3.2. Perlecan

Perlecan is another highly conserved HSPG of the ECM which contains multiple domains. The protein core consists of five domains sharing homology with other molecules of the nutrient metabolism, cell proliferation and adhesion and contains many binding sites for O-linked glycosylation and four sites for heparan sulfate and chondroitin sulfate chains attachment. The carbohydrates as well as the core protein are known to interact with a wide range of molecules to perform its functions [71].

PC plays an important role in development and organogenesis especially in cartilage and bone formation as well as in angiogenesis and wound healing. It has been proposed that PC regulates all these processes through its interactions with growth factors, morphogens and other matrix proteins. The particular local niche factors determine its function in every tissue [71]. 

In the cornea, PC is highly expressed in the BMZ of both cornea and limbus in infant and adult. The expression of PC has also been detected in Descemet’s membrane. Infant corneas have been reported to express perlecan in both faces of the Descemet’s membrane. However in the adult the expression of PC is restricted to the endothelial face [26]. 

PC might play a role in the maintenance of the cornea epithelium structure [72]. A study using a PC-deficient mice model shows that the epithelium is thinner and less differentiated in these mutants compared to wild type corneas and it is also accompanied by downregulation of Ki67, K12, Connexin 43 (Cx43), Notch1 and Pax6. However, the morphology is not altered in the mutant mice suggesting that PC is necessary for the cornea structure but it is not critical in its development.

#### 2.3.3. Versican

Versican is a large CSPG component of the ECM present in many different soft tissues. The structure of versican core protein consists of different domains, the globular aminno-terminal extreme (G1) binds to the glycosaminoglycan hyaluronan and the carboxyl-terminal extreme (G3) is a selectin-like domain consisting of a C-type lectin adjacent to two EGF domains and a complementary regulatory region. The middle region of the core protein is encoded by two large exons which contain the chondroitin sulfate regions of versican [73]. 

Research on versican has revealed a structural, biomechanical and cell biological function of this proteoglycan in different tissues both in development and disease. Particularly, it has been demonstrated both the anti- and pro-adhesive role of versican related to domains G1 and G3, respectively, suggesting that different products bound to versican can modulate cell adhesion in different ways and control functions such as cell proliferation and cell migration. It has been reported that versican can bind to cell surface receptors including integrins and cell adhesion molecules, and other ECM components, such as fibrillin and fibulin [73].

Versican plays a role in stem cell differentiation and maintenance. Versican might be involved in the differentiation process of cardiomyocytes from human embryonic stem cells [74]. In mice, versican might facilitate chondrocytes differentiation and regulates joint morphogenesis possibly via TGF-β signaling regulation [75]. In the cornea, versican has been most studied for its role during development [76]. Versican is highly expressed in rat cornea at early stages but decreases during development. At birth, versican is still highly expressed but it is undetectable at adulthood, suggesting its role in early stages of cornea development. A strong expression of versican in a band-like pattern closely associated with the BMZ at the anterior human limbus suggests a role of versican in limbal stem cell differentiation or migration [5].

An overall summary of the relative distribution of the ECM components is shown in Table 3. The ECM of the limbus is also composed by other weakly or moderately expressed components. These components include the glycoprotein clusterin and the proteoglycans endostatin, fibulin, and fibrillin. Immunoreactivity of all of these components has been observed in the BMZ of the limbus and co-localizated with some limbal stem cell markers, such as ABCG2 and p63. They may be involved in the normal homeostasis of the limbal stem cells. A better knowledge of these ECM components is necessary to confirm their role regulating limbal stem cells. 

**Table 3:** Expression of other ECM components in the basement membrane of adult human cornea, limbus and conjunctiva by immunohistochemistry.

ECM Components	Cornea	Limbus	Conjunctiva	Reference
Glycoproteins				
Fibronectin (FN)	w	+	w	[5]
Vitronectin (VN)	-/w	+	-/w	[5,39]
Tenascin C (TN-C)	-	+	-	[5,50]
SPARC/BM40	-	+	-	[5,26]
Nidogen 1	w	+	+	[5]
Nidogen 2	-/w	+	+	[5]
Thrombospondin 1 (TSP1)	+	-	-	[5]
Thrombospondin 4 (TSP4)	-/w	+	+	[5]
Proteoglycans				
Agrin	-/w	+	+	[5]
Perlecan (PC)	+	+	+	[5,26]
Versican	-	+	-	[5]

**+**: expression present; -: expression absent; w: weak expression; -/w: negative or weak expression

## 3. Conclusions

The interactions of the ECM components among each of them and with other cell surface molecules have been proposed to be essential in the limbal stem cell niche. They are part of the limbal structure and might function together with the signaling molecules involved in the maintenance of the stemness as well as regulate the limbal stem cells during normal homeostasis and wound healing. 

Further research is needed to identify the role of the different ECM components in the regulation of the limbal stem cells. This knowledge will be helpful in the bioengineering of an *in vitro* scaffold mimicking the limbal stem cell niche to expand these cells for therapeutic purpose. 
